# Ondasetron as Premedication for Unsedated Upper Digestive Endoscopy: A Cross‐Sectional Study Assessing Tolerance and Acceptability

**DOI:** 10.1002/hsr2.71853

**Published:** 2026-02-18

**Authors:** Espoir Batumike Murhi, Raissa Boroto Iranga, Corneille Lembembu, Tony Akilimali Shindano

**Affiliations:** ^1^ Faculty of Medicine Université Catholique de Bukavu Bukavu South Kivu Democratic Republic of Congo; ^2^ Hôpital Provincial Général de Référence de Bukavu (HPGRB) Université Catholique de Bukavu Bukavu South Kivu Democratic Republic of the Congo; ^3^ Regional School of Public Health Université Catholique de Bukavu Bukavu South Kivu Democratic Republic of Congo; ^4^ CTDGH, Centre for Tropical Diseases and Global Health Catholic University of Bukavu Bukavu South Kivu Democratic Republic of the Congo; ^5^ University of Kindu, Democratic Republic of the Congo Kindu Maniema Democratic Republic of the Congo; ^6^ Faculty of Medicine Université Officielle de Bukavu (UOB) Bukavu South Kivu Democratic Republic of the Congo

**Keywords:** acceptability, Democratic Republic of the Congo, gastroduodenal endoscopy, ondansetron, tolerance

## Abstract

**Background and Aims:**

In many low‐income countries, gastroduodenal endoscopy under conscious sedation remains uncommon due to added costs and concerns about managing complications and side effects. This study aimed to assess the feasibility of performing the procedure without sedation, using a potent antinauseant as premedication.

**Methodology:**

We conducted a prospective, single‐center study between 2020 and 2022 in Bukavu, eastern Democratic Republic of the Congo. Endoscopies were performed without sedation, supplemented by a protocol involving sublingual ondansetron spray. Clinical outcomes, procedural data, and patient feedback before and after the procedure were analyzed.

**Results:**

A total of 145 patients were enrolled, including 76 men (52.4%). Only 12 patients (8.28%) reported poor tolerance. The majority (91.72%) tolerated the procedure well, especially those who followed instructions and for whom endoscope insertion was straightforward (*p* < 0.001). Multivariate analysis identified ease of endoscope insertion as the primary determinant of tolerance (adjusted OR 44.34 [2.78–708.22], *p* = 0.007).

**Conclusion:**

Performing upper digestive endoscopy without sedation, but with ondansetron premedication, is generally well tolerated. Successful tolerance depends on thorough patient preparation and careful execution of the initial stages of the procedure.

## 1 Introduction

Gastroduodenal endoscopy (GDE) is among the most widely performed diagnostic procedures worldwide. It can be carried out by direct inspection or, more commonly today, with flexible video‐endoscopes. The technique enables both diagnosis and therapeutic intervention for major upper gastrointestinal diseases. Conscious sedation is increasingly used to enhance patient tolerance. Reports indicate that up to 5% of patients refuse the procedure without sedation, while 10% require significant persuasion to accept it under such conditions [1, 2, 3, 4].

Nevertheless, sedation carries risks and limitations. These include hypoxia, extended post‐procedure monitoring, increased staffing needs, higher costs, absence from work, and the requirement for an escort home. Adverse events occur in approximately 1 in 200 procedures, with 60% related to road accidents [2]. Some practitioners also argue that sedation may compromise diagnostic accuracy, particularly due to sedation‐induced gastro‐esophageal reflux [5, 6]. For these reasons, unsedated GDE remains the preferred approach in certain regions [7].

In Sub‐Saharan Africa, where universal healthcare coverage is lacking, endoscopy is frequently performed without sedation. Cost considerations are central, given the population's average income and the predominance of fee‐for‐service care [3].

Although tolerance and acceptance of unsedated GDE have been studied worldwide, data from the Democratic Republic of the Congo (DRC) are scarce. Endoscopy is relatively new in the country and limited to major cities due to shortages of equipment, trained personnel, and accessibility. A 2013 survey identified only 14 gastroenterologists nationwide (1 per 4 million inhabitants), compared with 1 per 14,000 in France; of these, only 4 were actively performing endoscopy [8].

In South Kivu province, endoscopy has been available at the Hôpital Provincial Général de Bukavu (HPGRB) since 2002. Since its introduction, the procedure has been surrounded by misinformation, rumors, and apprehension among the population. Some medical staff also hesitate to recommend it due to limited awareness. To date, no study has examined its sociological impact locally.

The present study aimed to assess the extent of reluctance, the underlying reasons for fear, and the degree of tolerance. We also sought to evaluate patient perceptions after the procedure and identify predictors of poor tolerance to improve practice. As a secondary objective, we investigated the potential benefit of ondansetron, a potent antinauseant, as premedication to enhance clinical tolerance.

## 2 Methodology

### 2.1 Study Design and Setting

We conducted a cross‐sectional study over 3 years (2020–2022) at HPGRB, a university hospital serving as the referral center for South Kivu province, which has a population of approximately 6 million. The province has two endoscopy units; the HPGRB unit is managed by a gastroenterologist assisted by a trainee intern.

### 2.2 Study Population

Eligible participants were patients undergoing diagnostic GDE without sedation. Exclusion criteria included pregnancy, age under 18 years, procedures performed under sedation or general anesthesia, therapeutic endoscopy, technical incidents (e.g., power failure, equipment malfunction), refusal to participate, or insufficient comprehension of the survey. Participants were continuously enrolled during the study period. The study was approved by the Ethical Committee of our hospital, and all patients enrolled gave their written informed consent to participate in the study.

### 2.3 Endoscopic Procedure and Data Collection

All procedures were performed by the same gastroenterologist using video endoscopy with an OLYMPUS multidirectional axial‐view fiberscope.

Prior to the procedure, the nurse provided psychological preparation and explained potential discomforts. Premedication consisted of sublingual ondansetron (8 mg spray, administered 15–30 min before) and oropharyngeal anesthesia with 2% Lidocaine spray. The choice of this formulation and route of administration (ondansetron oral spray) is related to the proven superiority of selective 5‐HT3 serotonin‐receptor antagonists over other conventional anti‐nausea drugs and the fact of their rapid absorption and quick reach of peak plasma concentration (10–15 min) at the standard dose of 8 mg in adults. Additionally, ondansetron has a significantly lower risk of severe cardiovascular and central nervous system side effects [9, 10].

Patients were positioned in the left lateral decubitus. The endoscope was introduced orally under direct vision, and the esophagus, stomach, and first two duodenal segments were examined. Five gastric biopsies were systematically obtained (two from the antrum, one from the angle, and two from the body).

Each patient completed a questionnaire before and after the procedure, covering:

- Pre‐procedure data: clinical history, last meal, prior information received, and apprehensions.
- Technical data: ease of endoscope insertion, cooperation, incidents, duration, and findings.
- Post‐procedure data: subjective tolerance and willingness to repeat the procedure under similar conditions.

**Figure 1:**
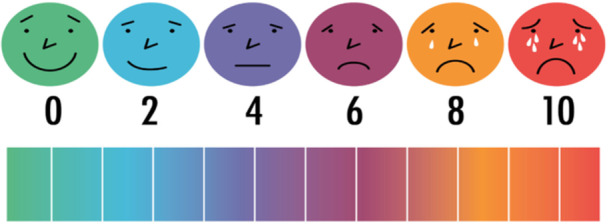
Endoscopy tolerance assessment tool.

Tolerance was assessed using a visual analog scale (endoscopy tolerance assessment tool, see Figure 1), with scores categorized as:

- Excellent tolerance: 0–2
- Acceptable tolerance: 3–4
- Low tolerance: 5–8
- Intolerance: > 8

### 2.4 Statistical Analysis

We used STATA 17 software (StatCorp) for data analyses. Quantitative variables were described by means and their standard deviations or medians and their interquartile ranges. The Kruskal–Wallis, Mann–Witney, and Student's *t*‐tests were used to assess the association between quantitative and categorical variables, as appropriate. Categorical variables were compared using the Chi‐square test and Fisher's exact test.

Simple logistic regression was performed by calculating the Odds ratio (OR) at its 95% confidence interval (CI). Differences observed were considered significant at a *p* < 0.05.

## 3 Results

**Figure 2:**
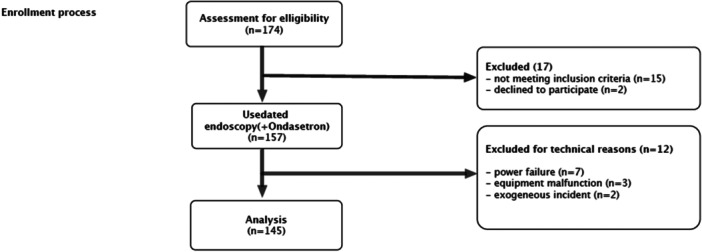
flowchart for patients' inclusion.

Of the 174 participants initially enrolled, only 145 took part in the study (Figure 2). Their general data according to tolerance are shown in Table 1. Epigastralgia was the main reason for consultation in about two‐thirds (69%). The most common diagnoses were gastritis (45.4%) and peptic ulcer disease (21.3%). Twelve percent had already had an endoscopy, while nearly half (47%) had never heard of it (Table 2). Only 12 patients (8.3%) said they didn't tolerate the procedure well, while most (91.7%) reported good or excellent tolerance. Age and gender did not seem to matter. A small amount of intolerance was seen among illiterate and university‐level patients. Those who tolerated the procedure best were the ones who followed instructions carefully and when the scope was easy to insert (*p* < 0.001). These patients were also much more willing to repeat the procedure compared with those who struggled (84.9% vs. 16.7%) [data not shown]. In multivariate analysis (Table 3), ease of insertion stood out as the main factor for good tolerance (adjusted OR 44.34 [2.78–708.22], *p* = 0.007).

**Table 1:** General characteristics of the study population.

Variables	Intolerance		*p* value
	No *n* = 133 (91.7%)	Yes *n* = 12 (8.13%)	
Median age	47 (18 80)	60 (18 80)	0.258
Sex			
Female (*n* = 69, 47.6%)	62 (46.62)	7 (58.33)	0.436
Male (*n* = 76, 52.4%)	71 (53.38)	5 (41.67)	
Level of education			
None	5 (3.76)	4 (33.33)	< 0.001
Primary	37 (27.82)	2 (16.67)	
Secondary	66 (49.62)	2 (16.67)	
University	25 (18.80)	4 (33.33)	
Height (cm)	161.57 ± 8.83	161.08 ± 8.33	0.854
Weight (Kgs)	63.60 ± 13.71	55.23 ± 8.64	0.040
BMI	24.41 ± 5.3	21.16 ± 1.9	0.039
Alcohol consumption	65 (48.87)	5 (41.67)	0.632
Smoking	20 (15.04)	2 (16.67)	0.573

**Table 2:** Univariate analysis of endoscopic procedure tolerance determinants.

Variables	Intolerance		
	No *n* = 133 (91.72%)	Yes *n* = 12 (8.27%)	*p* value
Endoscopy indications			
Epigastralgia	92 (69.17)	9 (75)	0.786
Digestive bleeding/Anemia	27 (20.30)	3 (25)	
Others	14 (10.53)	0 (0)	
Medical history and pre‐endoscopic information			
Previous endoscopy	18 (13.53)	0 (0)	0.190
Have received explanations about the procedure	43 (32.33)	4 (33.33)	0.586
Ever heard of endoscopy	72 (54.14)	4 (33.33)	0.140
Time interval since last meal (minutes)	808.50 (450–1200)	781.50 (30–1230)	0.563
Pre‐examination apprehensions			
Subjective anxiety	59 (44.36)	8 (66.67)	0.138
Degree of anxiety (using the tolerance assessment tool)	1.69 ± 2.05	2.91 ± 2.74	0.070
Fear of pain	40 (31.50)	5 (45.45)	0.264
Fear of nausea	4 (3.01)	1 (8.33)	0.355
Fear of vomiting	4 (3.01)	0 (0)	0.705
Fear of suffocation	4 (3 01)	0 (0)	0.705
Fear of injury	1 (0.7)	2 (16.67)	0.018
Endoscopy process			
Easy introduction	119 (89.47)	4 (33.33)	<0.001
Good cooperation (following instructions correctly)	119 (89.47)	5 (41.67)	<0.001

**Table 3:** Multivariate analysis of endoscopic procedure tolerance determinants.

Variables	OR adj (95% CI)	*f*
Weight	0.90 (0.78–1.04)	0.148
Level of education		
None	3.67 (0.03–463.52)	0.598
Primary	0.34 (0.01–8.29)	0.509
Secondary	0.01 (0.00–0.98)	0.049
University	1	
Fear of injury		
Yes	1	
No	5.97 (0.01–207413.80)	0.738
Easy introduction		
Yes	44.34 (2.78–708.22)	0.007
No		

## 4 Discussion

This study, done in a large hospital in South Kivu, showed that endoscopy was generally well tolerated (91.7%) even without sedation. Most patients were open to repeating the test under the same conditions. The key factor was how easily the scope could be inserted. In fact, 90.2% of patients had easy insertion. Most (84.9%) reported no unexpected symptoms, which gave them confidence for future exams. On average, the procedure lasted about 6 min, similar to sedated procedures. Some studies have found similar results [11, 12, 13]. This suggests that skipping sedation doesn't necessarily make the procedure longer if the doctor is experienced.

More than half of patients (52.7%) already knew about endoscopy, either because they had heard of it or had done it before (12.3%). Still, about a third (33.1%) were nervous, mainly afraid of pain. This shows that awareness of endoscopy is still limited in Sub‐Saharan Africa. In Morocco, though, almost half of patients had already had at least one endoscopy, showing higher familiarity [12].

Out of the 145 patients in our study, 68 (46.5%) felt anxious before the exam, mostly because they were worried about pain. This was lower than in Salwa's study, where nearly 78% of patients reported anxiety [12]. Only 4.1% of our patients experienced nausea, compared with almost 80% in Sombié's study [11]. The low rate of nausea here is probably thanks to our department's protocol, which combines Ondansetron with Lidocaine. Indeed, the addition of sublingual Ondasetron as premedication, a powerful anti‐nauseant, seems to have helped reduce this side effect.

We initially offered endoscopy to 147 patients, but two refused because of fear, giving us an acceptability rate of 98.6%. Of the 145 who went through with it, most (91.7%) tolerated the procedure well or found it acceptable. These results are very encouraging, showing tolerance levels close to those seen with sedation. For comparison, a study in Kinshasa (DRC) reported only 60% tolerance [13], while in Senegal, intolerance was seen in 42.5% of unsedated cases [14]. In Burkina Faso, tolerance was 84.6% [15], and in Iraq and Iran, rates were around 80% [16, 17]. Some Western data have reported tolerance rates of around 80% to 85% with conventional endoscopy without sedation [13, 14]. Nevertheless, better tolerance rates for procedures without sedation have been achieved with the use of ultrathin endoscopes via the transoral route or, better still, the transnasal route [18, 19, 20, 21, 22, 23].

What stands out is the importance of good preparation. Clear explanations of the procedure and possible discomforts make a big difference, as other studies have shown [24, 25, 26, 27, 28]. Patients in our study almost unanimously said the exam matched the explanations they were given beforehand, which likely boosted their confidence and tolerance.

The high tolerance we found may reflect both good practice and the effectiveness of our protocol. While sedation is usually recommended, our results suggest that adding Ondansetron can be a good alternative. It seems to make the insertion of the scope easier, which was the main factor linked to patient comfort. However, it should not be forgotten that easy insertion of the endoscope also depends on several other factors, including the operator's experience, the patient's level of preparation, and the anatomical characteristics specific to each patient.

Sedation is not common in our setting, mostly for financial reasons. Even so, the tolerance levels we achieved were similar to those in Western countries where sedation is standard, which suggests our approach is worth further evaluation.

Tolerance of the first endoscopy is important because it influences whether patients are willing to repeat the procedure. Sombié's study found similar acceptability rates (83.7% vs. 84.9%) [11]. In Morocco, however, only 43% of patients said they would repeat the exam under the same unsedated conditions [12].

It's also worth noting that patient comfort depends heavily on the operator's experience, and cultural factors play a role in how invasive procedures are accepted.

## 5 Study Limitations

This was a single‐center study, so the findings cannot be generalized to the whole Congolese or South Kivu population. Indeed, selection bias could occur due to the socio‐demographic and economic characteristics of the participants. We also didn't compare different premedication protocols (Lidocaine alone, Lidocaine + Ondansetron, or conscious sedation), which would have strengthened the conclusions. Another limitation is that the data came from only one operator, which could lead to a certain degree of subjectivity bias. Still, this is the first study to look at endoscopy acceptability in this community and the first to assess Ondansetron premedication. Finally, the study was conducted during the COVID‐19 pandemic, which may have reduced the number of procedures and influenced patient reactions.

## 6 Conclusion

Upper gastrointestinal endoscopy is still relatively new in many African countries, including the DRC. Its wider use is limited by patient fears and the challenges of sedation. Our study showed that endoscopy without sedation but with Ondansetron was well tolerated and comfortable for patients. Larger multicentric comparative studies (randomized controlled trial with stratification by drug dosages, patient groups, or endoscopy indications), including assessment of early or delayed adverse reactions, are needed to confirm these findings.

## Author Contributions

Espoir Batumike Murhi produced endoscopic data, assisted in data collection, and drafted the manuscript. Raissa Boroto Iranga, Corneille Lembembu, and Corneille Lembembu are responsible for data analysis. Tony Akilimali Shindano conceived and designed the study, produced endoscopic data, assisted in data analysis, and reviewed the manuscript. All authors have read and approved the final version of the manuscript.

## Funding

The authors received no specific funding for this work.

## Conflicts of Interest

The authors declare no conflicts of interest.

## Transparency Statement

The lead author, Tony Akilimali Shindano, affirms that this manuscript is an honest, accurate, and transparent account of the study being reported; that no important aspects of the study have been omitted; and that any discrepancies from the study as planned (and, if relevant, registered) have been explained.

## Data Availability

The datasets used and analyzed during the current study are openly available in Mendeley Data (Shindano, Tony (2024), “Ondasetron unsedated endoscopy” doi.10.17632/c6rygr549w.1).
